# Altered Gut Microbiota as an Auxiliary Diagnostic Indicator for Patients With Fracture-Related Infection

**DOI:** 10.3389/fmicb.2022.723791

**Published:** 2022-04-14

**Authors:** Xingqi Zhao, Wenli Tang, Haoyang Wan, Zixin Lan, Hanjun Qin, Qingrong Lin, Yanjun Hu, Guangchuang Yu, Nan Jiang, Bin Yu

**Affiliations:** ^1^Division of Orthopaedics and Traumatology, Department of Orthopaedics, Nanfang Hospital, Southern Medical University, Guangzhou, China; ^2^Guangdong Provincial Key Laboratory of Bone and Cartilage Regenerative Medicine, Nanfang Hospital, Southern Medical University, Guangzhou, China; ^3^Department of Bioinformatics, School of Basic Medical Sciences, Southern Medical University, Guangzhou, China; ^4^The Second Clinical Medical College, Southern Medical University, Guangzhou, China

**Keywords:** fracture-related infection (FRI), diagnosis, gut micobiota, dysbiosis index, short chain fatty acid

## Abstract

Preoperative diagnosis of fracture-related infection (FRI) is difficult for patients without obvious signs of infection. However, specific profiles of gut microbiota may be used as a potential diagnostic tool for FRI as suggested by a previous study. The fecal microbiome was compared between 20 FRI patients (FRI group), 18 fracture healed patients (FH group), and 12 healthy controls (HC group) included after collection of fecal samples and evaluation. The α and β diversity indices were used to characterize the fecal microbiome. Dysbiosis indexes were constructed based on the characteristic high-dimensional biomarkers identified in the fecal microbiota from the three groups by linear discriminant analysis and generalized linear model analysis to quantify the dysbiosis of fecal microbiota. The effectiveness of α and β diversity indices and dysbiosis indexes was assessed in distinguishing the fecal microbiome among the three groups. The influences of serum inflammatory factors on gut microbiota were also addressed. The α diversity indices were significantly different between the three groups, the highest in HC group and the lowest in FRI group (*P* < 0.05). The β diversity indices showed significant phylogenetic dissimilarity of gut microbiome composition among the three groups (*P* < 0.001). The dysbiosis indexes were significantly higher in FRI group than in FH and HC groups (*P* < 0.001). The area under Receiver operating characteristic curve showed the characteristics of gut microbiota and the gut microbiota was found as effective in distinguishing the three groups. The dysbiosis in the FRI patients was associated with systemic inflammatory factors. In addition, significant differences in the gut microbiota were not observed between the FRI patients versus without sinus tract or pus before operation. Since FRI patients, with or without sinus tract or pus, have a characteristic profile of gut microbiota, their gut microbiota may be used as an auxiliary diagnostic tool for suspected FRI.

## Introduction

Fracture-related infection (FRI) is one of the most challenging complications after orthopedic trauma surgery (Metsemakers et al., 2018). The average cost of a single treatment for FRI is more than US$15,000, four times that for periprosthetic joint infection (Morgenstern et al., 2018). Its treatment is difficult and its recurrence rate after therapeutic intervention is also very high, posing great pressure not only on its patients physically and mentally (Tseng et al., 2014) but also on their families and the medical insurance system (Hackett et al., 2015).

Early diagnosis of the disease is very important for the choice of treatment and recovery after surgery. However, when there are no obvious clinical signs of FRI, such as sinus tract or purulent discharge, diagnosis of FRI can only be made by intraoperative signs of infection, postoperative bacterial culture, and histopathological results (Sigmund et al., 2020). Therefore, it is likely to miss a diagnosis and thus conduct an inappropriate treatment in case of FRI, increasing the difficulty in treatment, chance of multiple operations, and recurrence rate due to undetected infection focus.

In early diagnosis of FRI, auxiliary detection of serum inflammatory factors is non-invasive, cheap, and capable of reflecting the body’s immune state quickly. However, their diagnostic values for FRI are limited due to their low sensitivity and specificity (Bosch et al., 2018; van den Kieboom et al., 2018; Sigmund et al., 2020).

Inflammatory diseases may also cause alterations in gut microbiota in addition to changes in serum inflammatory markers. Gut microbiota is coevolutionary with its host, affected by many host factors, such as age, diet, mood, medications, and other factors (Glassner et al., 2020). It can reflect growth, metabolism, and health status of the body (Aron-Wisnewsky et al., 2020). Previous studies have pointed out that the characteristics of gut microbiota in patients with irritable bowel syndrome (IBS) are significantly different from those in healthy people (Qin et al., 2010). The characteristic changes in gut microbiota were reported as a diagnostic marker of IBS (Zhou et al., 2018a). In addition, some scholars believed that the characteristics of gut microbiota might be used as a diagnostic tool for primary sclerosing cholangitis (PSC), a local inflammatory disease outside the intestine (Kummen et al., 2017; Rühlemann et al., 2017). Their results have suggested that the gut microbiota may serve as a diagnostic biomarker of PSC with a reasonable diagnostic accuracy differentiating PSC and healthy controls (Kummen et al., 2017).

McGinty and Mallon (2018) found that mice with reduced bone density exhibited characteristic changes in the gut microbiota. In addition, Levast et al. (2021) found that in patients with bone and joint infection, antibiotics altered the gut microbiota diversity and composition with only partial recovery, mucosal inflammation, and permeability and acquisition of multi-drug-resistant bacteria carriage. We reasonably speculate that FRI, an infectious inflammatory disease with destruction of bone structure and loss of bone mass after fracture or operation, may also cause changes in the gut microbiota in the process of infection and inflammation. Therefore, this study is to characterize the changes in gut microbiota in patients with FRI and to determine whether these changes may be specific enough to distinguish the FRI patients from their controls.

## Materials and Methods

### Study Population

In this observational case-control study, we recruited participants over a 6-month period between January 2019 and June 2019. They included patients with confirmed FRI (FRI group) from those who were scheduled to receive surgery for delayed fracture union at the Department of Orthopaedics and Traumatology, Nanfang Hospital, fracture healed patients (FH group) who were scheduled to remove the implants as fracture controls also at the Department of Orthopaedics and Traumatology, Nanfang Hospital, and healthy adults from the Health Examination Center at Nanfang Hospital as healthy controls (HC group).

### Ethical Approval

Informed consents conforming to the tenets of the Declaration of Helsinki were obtained from all participants prior to this study. The protocol of the study was approved by Medical Ethics Committee of Nanfang Hospital (NFEC-2019-087).

### Sample Collection

Fecal samples were collected in a sterile container (Cryogenic Vials, Corning, Lowell, MA, United States) within 24 h after admission, stored immediately into liquid nitrogen, and then transferred to a –80°C freezer until further analysis.

We first collected 72 stool samples from the patients suspected of FRI before operation. After evaluation by lab tests and self-administered questionnaire, only 20 patients were included for the present study and the other 52 were excluded to avoid influences of confounding factors such as, antibiotic or probiotic use in the past 2 weeks and other systemic or metabolic disease on gut microbiota. After the 20 FRI patients were determined, 18 fracture healed patients, and 12 healthy adults were included to match them in baseline data.

### Extraction of Genome DNA

Bacterial genomic DNA was extracted from fecal samples using a QIAamp DNA Mini Kit (Qiagen, Hilden, Germany) following the manufacturer’s specifications. DNA concentration and purity were monitored on 1% agarose gels. According to the concentration, DNA was diluted to 1 ng/μL using sterile water.

### Amplicon Generation

The barcoded primers 341F (CCTAYGGGRBGCASCAG) and 806R (GGACTACNNGGGTATCTAAT) were used to amplify the 16S rRNA gene V3 and V4 variable regions. PCR reactions were carried out in 30 μL reactions with 15 μL of Phusion^®^ High-Fidelity PCR Master Mix (New England Biolabs, Ipswich, MA, United States), 0.2 μM of barcoded primers, and 10 ng template DNA. Thermal cycling consisted of initial denaturation at 98°C for 1 min, followed by 30 cycles of denaturation at 98°C for 10 s, annealing at 50°C for 30 s, and elongation at 72°C for 30 s. For the final elongation step, 72°C for 5 min was used.

### PCR Products Mixing and Purification

After the same volume of 1 × loading buffer (SYB green contained) was mixed with PCR products, electrophoresis was performed on 2% agarose gel for detection. PCR products were mixed in equidensity ratios. Then, mixture PCR products were purified with GeneJET™ Gel Extraction Kit (Thermo Scientific, Carlsbad, CA, United States) according to the manufacturer’s protocol.

### Library Preparation and Sequencing

Sequencing libraries were generated using Ion Plus Fragment Library Kit 48 rxns (Thermo Scientific, Guilford, CT, United States) following manufacturer’s recommendations. The library quality was assessed on the Qubit@2.0 Fluorometer (Thermo Scientific, Shanghai, China). At last, the library was sequenced on an Ion S5™XL platform and 400/600 bp single-end reads were generated.

### Bioinformatics Processing

Sequence reads were preprocessed on QIIME2 workflow (Bolyen et al., 2019). After each sample was demultiplexed and denoised by DADA2 (with parameters: –p-trim-left 0 –p-max-ee 2 –p-trunc-q 2 –p-trunc-len 200) (Callahan et al., 2016), the clean data were annotated by RDP against GreenGenes Database (v13_8) (DeSantis et al., 2006), followed by construction of an amplicon sequence variant (ASV) table, based on which a phylogenetic tree was created by PyNAST (Caporaso et al., 2010a) and FastTree (Price et al., 2009), and then α and β diversities were calculated. In the present study, α diversity was indicated by the Shannon index which shows the number and distribution of microbial species in a sample and the phylogenetic distance (PD) whole tree index which shows the range of PDs among microbial species. The β diversity here was indicated by UniFrac distance which illustrates the phylogenetic dissimilarity among samples. A smaller UniFrac distance between two samples indicates a higher similarity. The distances were calculated and compared by principal coordinate analysis (PCoA) and permutational multivariate analysis of variance (PERMANOVA) to determine the differences in bacteria composition among the three groups. Linear discriminant analysis effect size (LEfSe) and generalized linear model (GLM) were used to identify the enriched bacteria for each group according to which dysbiosis index was obtained to distinguish FRI group from controls (HC and FH groups).

Considering the restriction of DADA2 at selecting ASVs, we also performed a closed-reference operational taxonomic units (OTU) picking method by SortMeRNA (Kopylova et al., 2012) on QIIME 1.9.1 (Caporaso et al., 2010b), and constructed an OTU table for subsequent analysis. A dysbiosis index was also constructed and evaluated as described above.

### Statistical Analysis

Statistical analyses were made using R (v4.1.1). The current study used Wilcoxon rank sum test to identify differences between the two groups, and used the Chi-squared test, Analysis of Variance (ANOVA) test, or Kruskal Wallis test for the three groups according to data type. Logistic regression was used to evaluate the odd ratio of serum inflammatory factors by R package *forestplot* (v2.0.0). Spearman correlation analysis by R package *corrplot* (v0.90) found the correlation between serum inflammatory factors and ASVs. Receiver operating characteristic (ROC curves) and area under the curve (AUC) were performed using R package *pROC* (v1.17.0.1) to evaluate classification effect.

## Results

### Clinical Characteristics of Included Participants

The clinical characteristics of the 20 FRI patients, 18 FH patients, and 12 HC are shown in Table 1. The sex ratio between the three groups showed no significant difference (χ^2^ = 2.039, *P* = 0.361, Chi-squared test). In addition, no significant difference was found either regarding the mean age between the three groups (*F* = 1.189, *P* = 0.313, ANOVA test). In FRI group, three patients (15%) had a sinus tract, and 13 patients (65%) had a purulent exudation preoperatively. The remaining four patients were diagnosed by intraoperative signs of pus or microbial growth in at least two samples, or >5 neutrophil polymorphs per high power field in the histopathological analysis postoperatively (Table 1).

**TABLE 1:** Clinical characteristics of the included participants.

	HC (*n* = 12)	FH (*n* = 18)	FRI (*n* = 20)	*P* value
**Demographics**				
Gender, M/F	7/5	14/4	16/4	0.361^†^
Mean age, years (range)	37.83 (23–62)	40.78 (11–68)	46.05 (13–73)	0.313*
**Site of fracture, *n* (%)**				
Tibia		5 (27.78%)	12 (60.00%)	
Tibia and fibula		1 (5.56%)	3 (15.00%)	
Patella		1 (5.56%)	0	
Femur		2 (11.11%)	1 (5.00%)	
Humerus		3 (16.67%)	0	
Radius and/or ulna		3 (16.67%)	0	
Clavicle		1 (5.56%)	0	
Foot and ankle		1 (5.56%)	3 (15.00%)	
Knee joint		0	1 (5.00%)	
Elbow joint		1 (5.56%)	0	
**Preoperative local status, *n* (%)**				
Sinus tract or fistula		0	3	
Visible pus		0	13	

HC, healthy controls; FH, fracture healed; FRI, fracture-related infection; M/F, male/female. ^†^Chi-squared test. *ANOVA test.

### The Gut Microbiota Composition in Fracture-Related Infection, Fracture Healed, and Healthy Controls Groups

**FIGURE 1:**
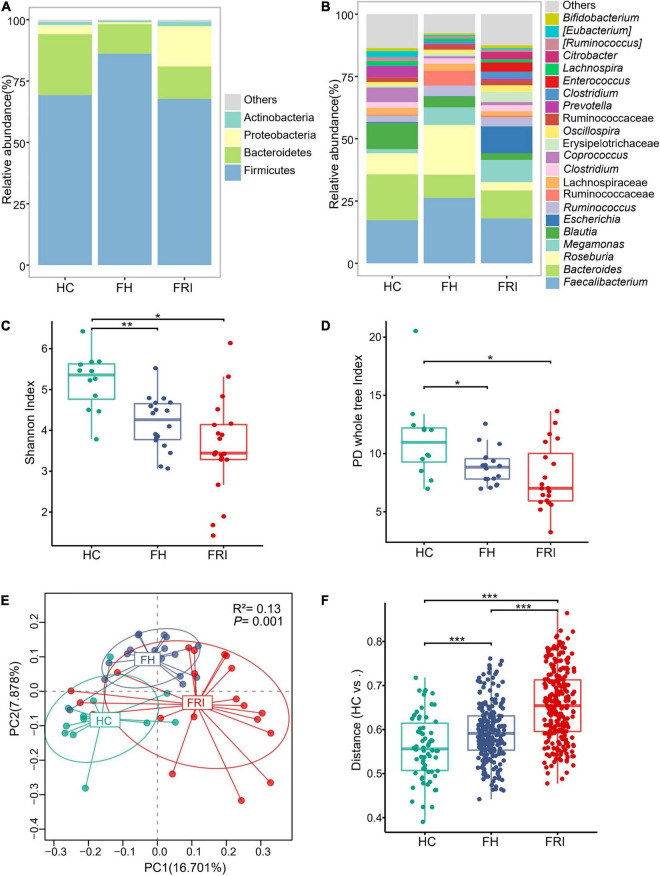
The gut microbiota composition in fracture-related infection (FRI), fracture healed (FH), and healthy controls (HC) groups. **(A)** Taxonomy composition at the phylum level. **(B)** Taxonomy composition at the genus level. **(C)** α Diversity indicted by Shannon’s diversity index. **(D)** α Diversity indicated by PD whole tree index. **(E)** Principal coordinate analysis (PCoA) based on Unweighted UniFrac distance. **(F)** Comparisons of β diversity indicated by Unweighted UniFrac distance between samples in HC group and each group. “Others” represents lower-abundance taxa. *P < 0.05, **P < 0.01, and ***P < 0.001.

We analyzed the fecal samples from the 50 participants for taxonomic composition and differential abundance of their gut microbiota. The gut microbiota from all participants was composed mainly of the phylum *Firmicutes*, the phylum *Bacteroidetes* and the phylum *Proteobacteria*. Rough observation showed that the main component was *Bacteroidetes* in HC group, *Firmicutes* in FH group, and *Proteobacteria* in FRI group (Figure 1A and Supplementary Figure 1A). The FRI group showed a higher abundance of the genus *Escherichia* and a lower abundance of the genus *Roseburia*, which was opposite of the FH group (Figure 1B and Supplementary Figure 1B).

The microbial community richness and evenness indicated by Shannon index and diversity indicated by PD whole tree index both showed a marked decrease in the FRI group (Figures 1C,D). PCoA and PERMANOVA analyses based on Unweighted UniFrac distance displayed that the overall composition of the gut microbiota was significantly different among the three groups (Figure 1E), and the difference between FRI and HC groups was greater than that between FH and HC groups (Figure 1F).

### Bacterial Differential Abundance in Fracture-Related Infection, Fracture Healed, and Healthy Controls Groups

**FIGURE 2:**
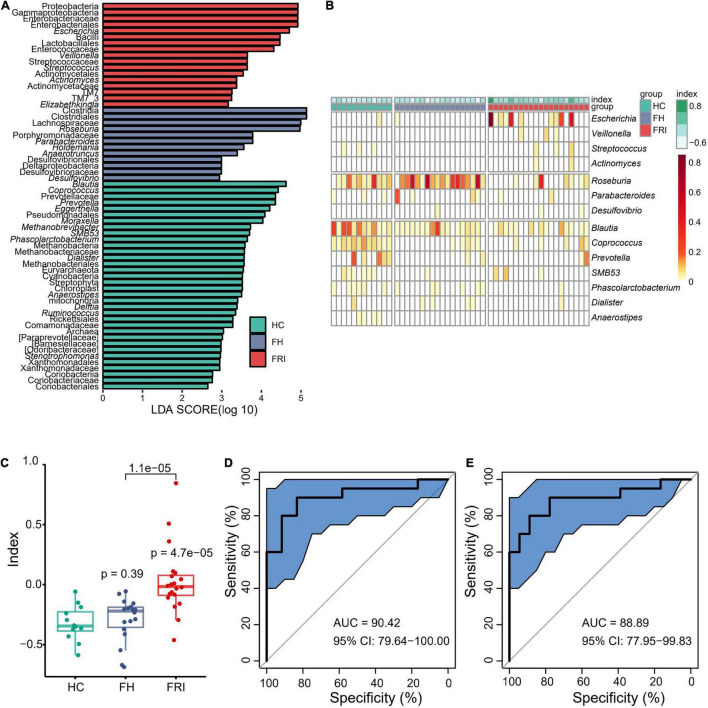
Bacterial differential abundance in FRI, FH, and HC groups. **(A)** LDA score of Linear discriminant analysis effect size (LEfSe) (FRI vs. FH vs. HC). **(B)** Bacteria genera for dysbiosis index building. **(C)** Dysbiosis Index (FRI vs. FH vs. HC). **(D)** Area under the curve (AUC) showing diagnostic accuracy of gut microbiota composition for FRI vs. HC. **(E)** AUC showing diagnostic accuracy of gut microbiota composition for FRI vs. FH.

To further define the differences in microbiota between FRI and FH groups and look for high-dimensional biomarkers for FRI, LEfSe was performed to calculate a linear discriminant analysis value (LDA score, Figure 2A and Supplementary Figure 2). In comparison with FH group, the fecal microbiota in FRI group was characterized by a higher abundance of *Gammaproteobacteria, Proteobacteria, Enterobacteriaceae, Escherichia, Bacilli*, *Lactobacillales, Pseudomonadales, Streptococcaceae, Streptococcus* and *Actinomycetales*, but a lower abundance of *Clostridiales, Clostridia, Lachnospiraceae, Roseburia, Anaerotruncus*, and *Parabacteroide* (Figure 2A and Supplementary Figures 2, 3).

FRI group showed a reduced bacterial diversity with obvious dysbiosis. We removed those with low relative abundance and prevalence to select differential genera (<0.01% in ≥20% of samples) which were then used to calculate a dysbiosis index by the formula: $\frac{\sum {\text{RelativeAbundance}}_{\mathrm{enriched}\mathrm{in}\mathrm{FRI}}}{\sum {\text{RelativeAbundance}}_{\mathrm{enriched}\mathrm{in}\mathrm{FH}\mathrm{or}\mathrm{HC}}}$. Finally, 14 genera were used: *Escherichia, Veillonella, Streptococcus*, *Actinomyces*, *Roseburia*, *Parabacteroides*, *Desulfovibrio*, *Blautia*, *Coprococcus*, *Prevotella*, *SMB53*, *Phascolarctobacterium*, *Dialister*, and *Anaerostipes*. The dysbiosis index was significantly higher in FRI group than in FH and HC groups (Figures 2B,C and Supplementary Figure 3). Moreover, there was no significant difference between FH and HC groups (*P* = 0.39), further indicating that a local bone infection might cause changes in gut microbiota (Figure 2C). In addition, FRI group was effectively distinguishable from HC and FH groups in gut microbiota composition as a differential diagnostic index (Figures 2D,E).

The present study also conducted a more rigorous filter process using GLM analysis taking into account age and gender in order to ensure the reliability of the selected genera. The filter process included: (1) Differential Genus detection based on comparisons of FRI vs. FH vs. HC, FRI vs. HC, and FRI vs. FH. Genus with a Pr (>|z|)value < 0.05 were considered as significantly Differential Genus. (2) Taking the union of the above-mentioned differential Genus in each comparison for the dysbiosis index construction. The comparison of differences between cohorts based on the newly constructed dysbiosis index was consistent with the results of above mentioned 14 selected differential genera based on LEfSe analysis (Supplementary Figure 4).

Corresponding to the results of DADA2 preprocessing, the differential Genus identified by both LEfSe and GLM methods using data preprocessed by closed-reference method were highly consistent with DADA2. The constructed dysbiosis index can also discriminate FRI patients from controls (Supplementary Figures 5, 6).

### Fecal Microbiota Composition in Relation to Visible Pus or Sinus Tract in Fracture-Related Infection Group

**FIGURE 3:**
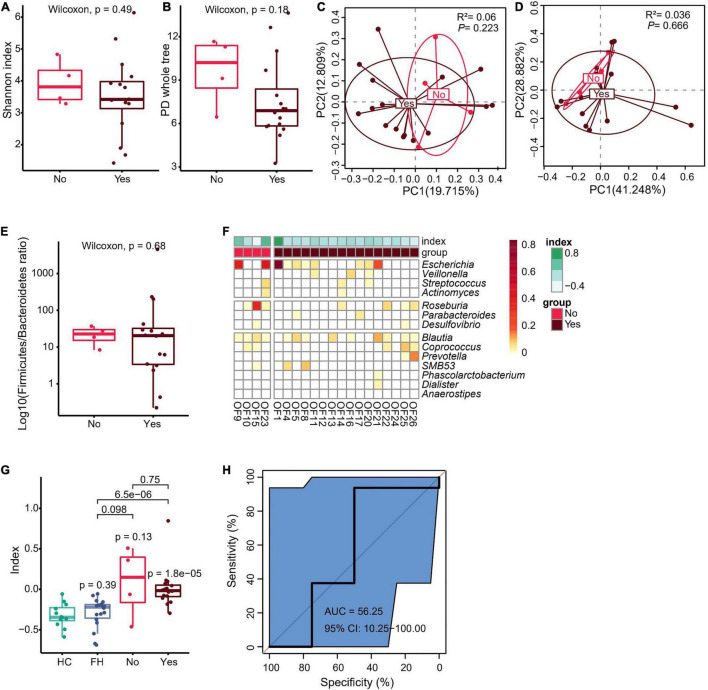
Fecal microbiota composition in relation to visible pus or sinus tract in FRI group. **(A)** α Diversity indicated by Shannon’s diversity index. **(B)** α Diversity indicated by PD whole tree index. **(C)** PCoA based on Unweighted UniFrac distance. **(D)** PCoA based on weighted UniFrac distance. **(E)** The Firmicutes to Bacteroidetes ratio. **(F)** Bacteria genera for dysbiosis index building. **(G)** The dysbiosis index (HC vs. FH vs. Yes vs. No). **(H)** AUC showing diagnostic accuracy in judgment of sinus tract or pus before operation in FRI group based on gut microbiota composition (Yes vs. No). “Yes” for FRI subgroup with sinus tract or pus before operation and “No” for FRI subgroup without sinus tract or pus before operation.

In order to clarify whether preoperative sinus tract or pus might have affected the gut microbiota, the current study further divided FRI group into two subgroups: those with sinus tract or pus before operation versus those without sinus tract or pus before operation. There was no significant difference in fecal microbiota richness and diversity, Firmicutes to Bacteroidetes ratio, and dysbiosis index between the two groups (Figures 3A–G and Supplementary Figures 5–7A,B). Moreover, the results of AUC showed that microbiota composition was not effectively distinguishable between the two groups (Figure 3H and Supplementary Figures 4G, 5, 6). However, when microbiota composition was used as a differential diagnostic index, the effectiveness of differential diagnosis was higher in comparisons between HC group and FRI subgroup without sinus tract or pus before operation (Supplementary Figure 7C), between HC group and FRI subgroup with sinus tract or pus before operation (Supplementary Figure 7D), between FH group and FRI subgroup without sinus tract or pus before operation (Supplementary Figure 7E), and between FH group and FRI subgroup with sinus tract or pus before operation (Supplementary Figure 7F) than in the comparison between FRI subgroups with and without sinus tract or pus preoperatively (Figure 3H and Supplementary Figures 4G–K, 7C–F). In addition, the Wilcoxon rank sum test showed no significant differences in the relative abundance of the FRI-related taxa between the two subgroups (Figures 3F,G and Supplementary Figure 4C). All these results indicated that the presence of sinus tract or pus before operation might not have significantly affected the gut microbiota from FRI group.

### Fecal Microbiota Composition in Relation to Serum Inflammatory Markers

**FIGURE 4:**
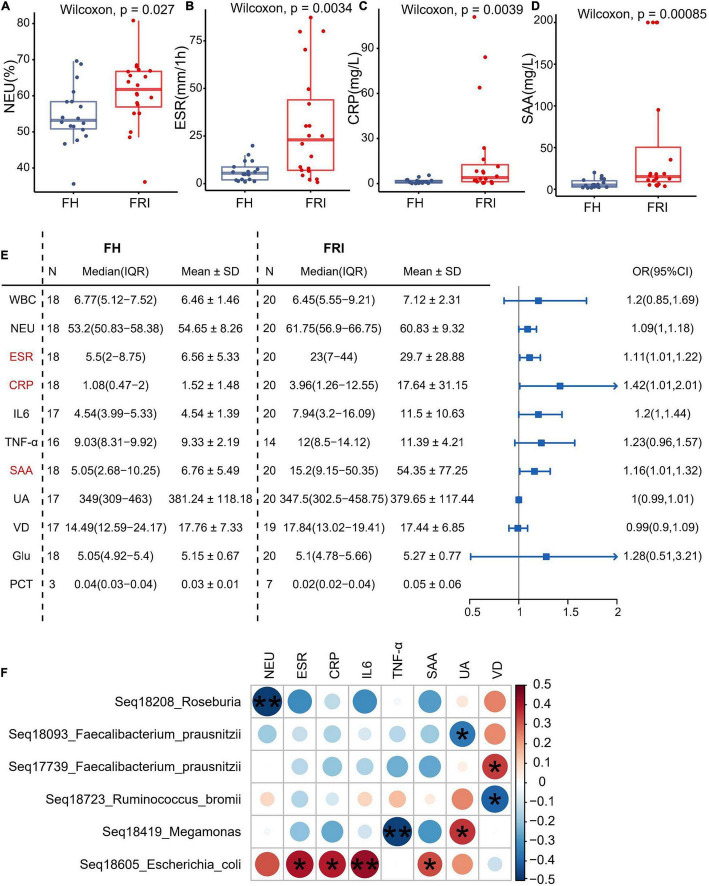
Fecal microbiota composition in relation to serum inflammatory markers. **(A–D)** Levels of serum inflammatory markers in FRI and FH groups. **(E)** Odds ratio (OR) values of serum inflammatory markers in FRI group compared to FH group. **(F)** Bubble chart showing significantly correlations between bacterial amplicon sequence variants (ASVs) and serum inflammatory markers. NEU, percentage of neutrophils; ESR, erythrocyte sedimentation rate; CRP, C-reactive protein; SAA, serum amyloid A; WBC, white blood cell count; IL-6, interleukin-6; TNF-α, tumor necrosis factor-α; UA, uric acid; VD, vitamin D; Glu, glucose; PCT: procalcitonin. *P < 0.05, and **P < 0.01.

In order to explore the possible association between serum inflammatory markers and changes in the gut microbiota composition, the current study observed the serum inflammatory markers in FRI and FH groups. The levels of neutrophils, erythrocyte sedimentation rate (ESR), serum C-reactive protein (CRP) and serum amyloid A (SAA) were significantly higher in FRI group than in FH group (Figures 4A–D and Supplementary Figure 8), and the odds ratio (OR) was greater than 1 in ESR, serum level of CRP and SAA (Figure 4E). When the serum inflammatory markers were used as differential diagnosis indicators, their greatest AUC was 81.81%, indicating they were not as effective as the composition of gut microbiota for differential diagnosis of FRI (Figures 2D,E and Supplementary Figure 9). In addition, the Spearman correlation analysis showed that the relative abundance in the FRI-related taxa identified above was associated with rise of serum inflammatory markers (Figure 4F). All these indicated that changes in the gut microbiota composition might have been associated with the increased systemic inflammatory markers during FRI.

## Discussion

In the present study, we conducted an observational case-control study to examine the profiles of gut microbiota in FRI and FH patients and healthy volunteers and to explore its value as an auxiliary diagnostic tool. We found that FRI patients showed reduced bacterial diversity with obvious dysbiosis and that the characteristic gut microbiota was effective enough to distinguish FRI patients from FH and HC. Furthermore, the dysbiosis indexes were higher in FRI patients than in FH patients. This dysbiosis of FRI, at least partially, was induced by changes in systemic inflammatory factors caused by local infection. In addition, presence of sinus tract or pus before operation did not affect significantly the gut microbiota of FRI patients.

Although the studies regarding gut microbiota as a diagnostic tool were mostly concerned with the diseases related to systemic, metabolic, or digestive system, little attention has been paid to changes in gut microbiota caused by local extraintestinal lesions. A recent study showing that ischemic stroke caused changes in the composition of gut microbiota (Xu et al., 2021) confirmed that the stroke, as the initial factor, induced dysbiosis of gut microbiota. We assumed that FRI, as an inflammatory reaction to persistent infection of bone tissue, might cause a similar immune response and lead to corresponding changes in gut microbiota.

The present study found a higher abundance of the phylum *Proteobacteria*, especially the class *Gammaproteobacteria*, the family *Enterobacteriaceae*, and the genus *Escherichia* in FRI group compared with FH controls (Figures 1, 2 and Supplementary Figures 1–3). *Proteobacteria* are a phylum, consisting of Gram-negative staining bacteria containing proinflammatory lipopolysaccharides (LPS) in their cell membrane, which is overrepresented in the gut in several conditions characterized by chronic inflammation (Rizzatti et al., 2017). The family *Enterobacteriaceae*, belonging to the *Gammaproteobacteria*, have been found to be enriched in the gut in inflammatory bowel disease (IBD) (Kostic et al., 2014) and PSC (Alberts et al., 2018). A recent study has shown the elevated abundance of *Enterobacteriaceae* in the gut of stroke patients. At the same time, excessive proliferation of *Enterobacteriaceae* in the intestine, in turn, affected the recovery of stroke (Xu et al., 2021). The growth of *Enterobacteriaceae* in the intestines along with the impaired gut barrier rapidly induced systemic inflammation through the production of a large amount of LPS. *Escherichia coli*, belonging to the family of *Enterobacteriaceae*, which can persist and replicate inside epithelial cells and macrophages, were increased in the ileal mucosa in IBD (McIlroy et al., 2018). Presence of adherent and invasive bacteria, mainly *Escherichia coli*, was also reported in ankylosing spondylitis in association with gut inflammation and damage to the intestinal mucosal barrier (Ciccia et al., 2017). The order *Pseudomonadales* also belongs to the class *Gammaproteobacteria* and to the phylum *Proteobacteria*. An increase in the *Gammaproteobacteria* and *Pseudomonas* was also shown in reactive arthritis (Manasson et al., 2018).

*Streptococcus* are a genus of coccus Gram-positive bacteria belonging to the phylum *Firmicutes*, the class *Bacilli*, and the order *Lactobacillales*. The FRI patients in the current study showed an increase in the genus *Streptococcus*. Also, *Staphylococcus aureus* was still the main pathogen causing FRI (Wang et al., 2021). Interestingly, a previous study stratified the common pathogenic microorganisms of FRI into low virulence (Coagulase negative *Staphylococci*, *Bacillus*) and high virulence (*Staphylococcus aureus*, *Streptococci*, *Enterococci*, and *Enterobacteriaceae*) pathogens (Sigmund et al., 2020). Although the abundance of most of these pathogenic microorganisms mentioned above was up-regulated in the gut microbiota, we are not sure whether the corresponding up-regulation of bacterial abundance in the intestinal tract was caused by the bacterial translocation from the bone tissue focus in FRI patients. However, in our previous study of animal osteomyelitis, the results showed that OTU numbers of *Staphylococcus* and *Lysobacter* were higher and that of *Akkermansia* was significantly lower in the mice with osteomyelitis than in the mice without osteomyelitis but only with an intramedullary nail (Zhao et al., 2021). In addition, a gain in *Streptococcus* in fecal samples has also been found in new-onset Crohn’s Disease (CD) and has been associated with a higher recurrence of CD after surgery (Gevers et al., 2014; Pascal et al., 2017; Zhou et al., 2018b).

The FRI patients showed a relative decrease in the class *Clostridia*, the order *Clostridiales*, the family *Lachnospiraceae*, and the genus *Roseburia* compared with FH controls (Figures 1, 2 and Supplementary Figures 1–3). A decreased level of *Clostridiales* was also found in patients with IBD and its low abundance was associated with a higher recurrence of CD after surgery and a poorer outcome after treatment with infliximab (Sokol et al., 2008). The order *Clostridiales* has been shown to have immune-shaping effects (Sun et al., 2021), which is attributed to the short-chain fatty acids (SCFAs), including acetate, propionate, and butyrate (Pasztoi et al., 2015; Tsukuda et al., 2021). A series of studies have pointed out that administration of SCFAs contributed to long-term radioprotection, mitigation of hematopoietic and gastrointestinal syndromes, a reduction in proinflammatory response (Guo et al., 2020), and an increased number of colonic Tregs (Pasztoi et al., 2015). Besides, supplement of SCFAs producing bacteria has been found to produce a protein which inhibits the NF-κÂ pathway, to stimulate production of IL-10 and to be able to inhibit experimental colitis in mice (Sokol et al., 2008). In addition, SCFAs exert protective effects against enteric pathogen colonization and infection through multiple mechanisms and can act to regulate virulence in different pathogens (Sun and O’Riordan, 2013). The family *Lachnospiraceae* and the genus *Roseburia* are also responsible for SCFAs production (Zheng et al., 2020). Elevated abundance of members of the family *Lachnospiraceae* was associated with postradiation restoration of hematopoiesis and gastrointestinal repair (Guo et al., 2020). An earlier study on the fecal microbiota in children with enthesitis-related arthritis reported findings, similar to the present study, that abundance of the family *Lachnospiraceae* was lower among the patients (Stoll et al., 2014). A recent study also showed a reduction in *Lachnospiraceae* in postinfectious spondyloarthritis (Manasson et al., 2018).

**FIGURE 5:**
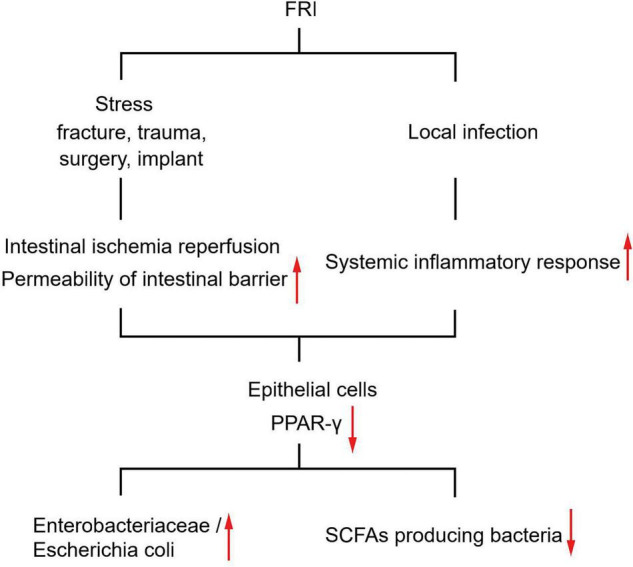
Diagram of proposed mechanisms for gut microbiota changes induced by FRI.

Although it is elusive how FRI might have caused the above changes in gut microbiota, several prevailing hypotheses may explain the link. Previous studies have shown that stress factors such as fracture trauma, operation process, and infection might cause intestinal ischemia-reperfusion, affect the intestinal epithelial cell layer and mucosal layer, and increase the permeability of intestinal barrier (Napolitano et al., 1996; Levy et al., 2006; Wrba et al., 2019). The intestinal ischemia-reperfusion could finally drive the gut dysbiosis due to nitrate respiration of the gut microbiota (Xu et al., 2021). Recently, some researchers have found that intestinal permeability increases significantly after modeling fractures in mice, and *Akkermansia* supplementation can repair intestinal mucosa and promote fracture healing (Liu et al., 2020). Thus, we speculate that the change in gut microbiota caused by FRI might be realized through stress and subsequent changes in intestinal permeability. In addition, although FRI is a local infection, it can cause a systemic inflammatory response. In fact, the levels of serum inflammatory markers were significantly higher in FRI patients than in FH patients. As is shown in Figure 4F, levels of serum inflammatory markers were correlated positively with abundance of *Escherichia coli* and negatively with abundance of *Roseburia*. This may also be one of the reasons for the change in gut microbiota. As for the specific molecular mechanisms, studies have shown that stress and inflammation can cause inhibition of the PPAR-γ pathway (peroxisome proliferator-activated receptor-gamma), as in osteoarthritis (Zhu et al., 2019; Tian et al., 2021). Previous studies have shown that inhibition of PPAR-γ pathway in intestinal epithelial cells leads to an increase of *Enterobacteriaceae*/*Escherichia coli* in gut microbiota and a decrease of butyrate producing bacteria (*Clostridia* and *Lachnospiraceae*), a change similar to that of gut microbiota in FRI patients (Figure 5). However, the detailed mechanisms how the PPAR-γ pathway acts in the gut and its associated metabolism deserves further study on FRI.

A large proportion (16/20, 80%) of the FRI patients included in the current study had visible pus or sinus at the outpatient department. However, for those who showed no obvious infection symptoms, a preoperative diagnosis was still difficult. Therefore, the current study compared their gut microbiota despite sinus tract and visible pus. The results showed no significant difference in microbiota composition, diversity, or dysbiosis index between the two subgroups. In addition, the two subgroups were not distinguishable using microbiota composition as a differential diagnostic index. All these results indicate that their gut microbiota might not have been affected by presence or absence of sinus tract or pus, but rather by presence of FRI itself.

A major limitation of the study was the sample size. The current study included some pediatric patients (age less than 18), and although there was no gender difference, there were more male patients. Such imbalances in sample collection may affect the results. Thus, the current findings need further validation in a larger cohort. Also, the present group of patients could not demonstrate any possible differences in gut microbiota between aseptic non-union and aseptic non-union after fracture. It could not be ignored that factors such as diet, geography, ethnicity, and other host factors have a potential impact on the gut microbiota of the host (Yatsunenko et al., 2012). Therefore, our present conclusion should be interpreted with caution and that the gut microbiota of FRI patients may serve as a differential biomarker for FRI. It is certain that further research is required.

In summary, the results of present study demonstrated significant compositional variations in gut microbial communities in FRI patients compared with FH and HC. The results showed that FRI might result in characteristic gut microbiota despite sinus tract or pus compared with FH and HC. We thus concluded that the gut microbiota might be used as an auxiliary diagnostic tool for suspected FRI patients. The clinical value of gut microbiota for FRI can be established only after its sensitivity and specificity have been fully explored in further studies.

## Data Availability Statement

The sequencing data have been deposited at the European Nucleotide Archive (ENA) database under accession no. PRJEB45380. Further information and requests for resources and reagents should be directed to and will be fulfilled by the corresponding author. The main R scripts of the current manuscript have been deposited at GitHub (https://github.com/WENLITANG/FRI_GutMicrobiota_analysis).

## Ethics Statement

All participants in the study read and signed informed consent, and the Research Ethics Committee at Nanfang Hospital, Southern Medical University approved the study protocol (NFEC-2019-087).

## Author Contributions

XZ, WT, GY, and BY: conception and design. XZ, HW, HQ, and ZL: sample collection. XZ, HW, HQ, ZL, and NJ: acquisition of data. XZ, NJ, QL, and YH: processing of specimens and generation of data. XZ, WT, GY, NJ, QL, and YH: analysis and interpretation of data. XZ, WT, HW, and BY: drafting or revising the manuscript. GY, NJ, and BY: final approval of the manuscript. GY and BY: access to all study data and takes responsibility for the data integrity and accuracy. All authors read and approved the final manuscript.

## Conflict of Interest

The authors declare that the research was conducted in the absence of any commercial or financial relationships that could be construed as a potential conflict of interest.

## Publisher’s Note

All claims expressed in this article are solely those of the authors and do not necessarily represent those of their affiliated organizations, or those of the publisher, the editors and the reviewers. Any product that may be evaluated in this article, or claim that may be made by its manufacturer, is not guaranteed or endorsed by the publisher.
