# Supplementary figures and images for: Altered Gut Microbiota as an Auxiliary Diagnostic Indicator for Patients With Fracture-Related Infection
Source: Front Microbiol. 2022 Apr 14;13:723791. doi: 10.3389/fmicb.2022.723791 (PMC9048737; doi:10.3389/fmicb.2022.723791)

a

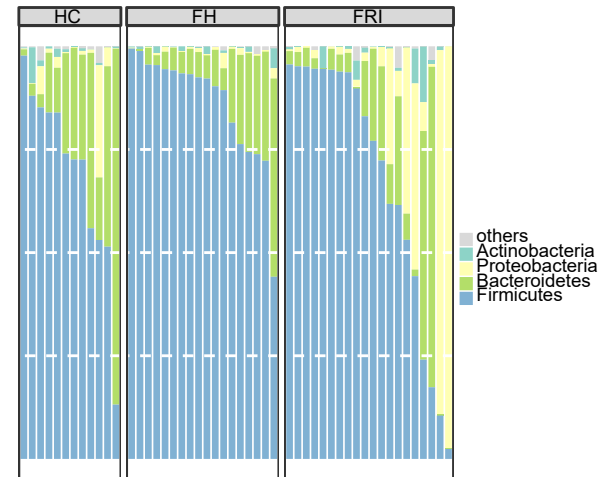

b

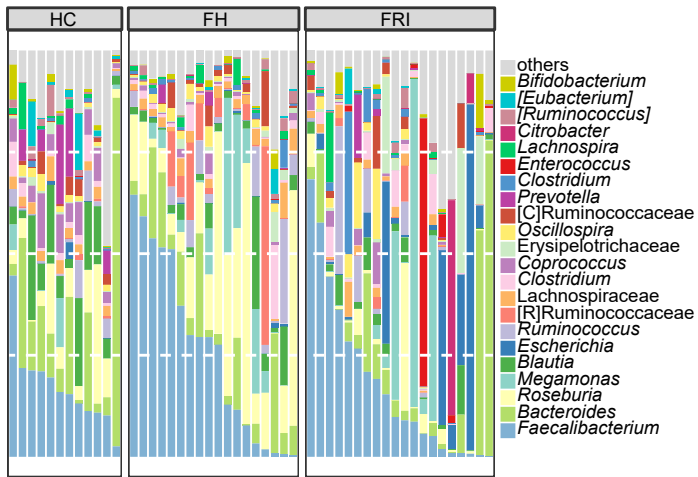

c

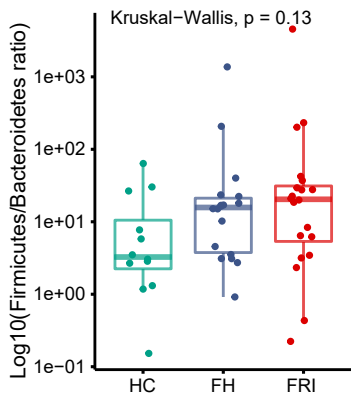

Supplement: Supplementary file 1 [file Image_1.PDF]

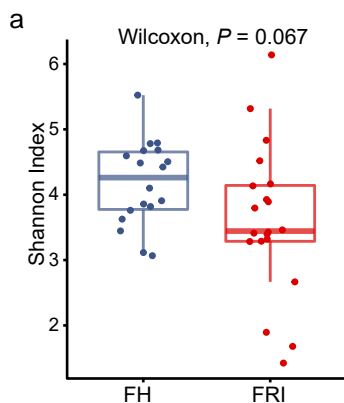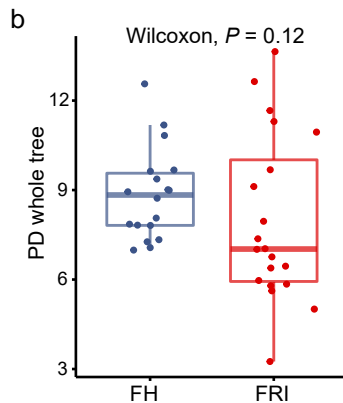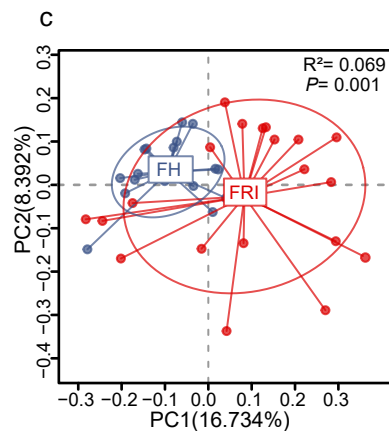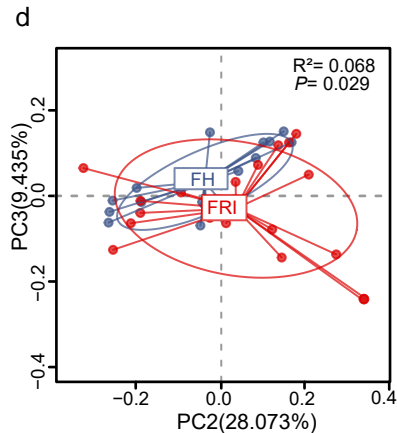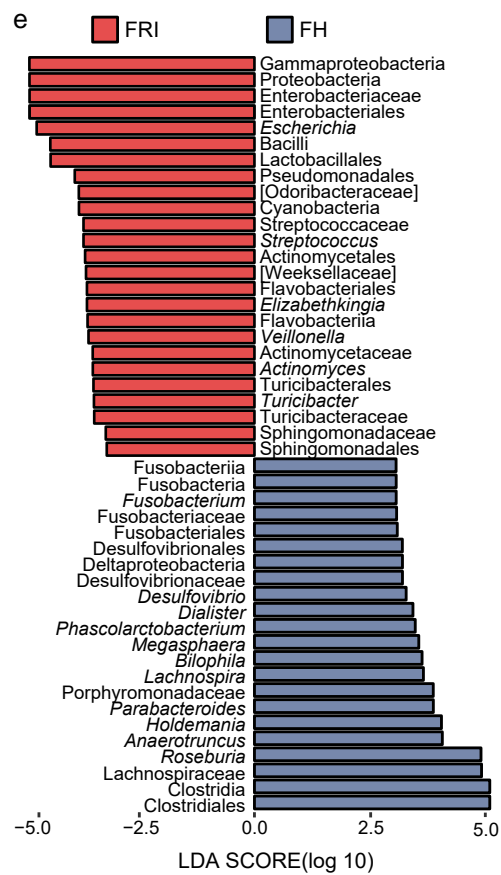

Supplement: Supplementary file 2 [file Image_2.PDF]

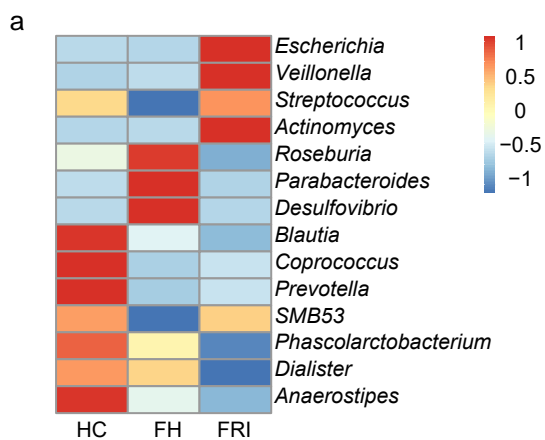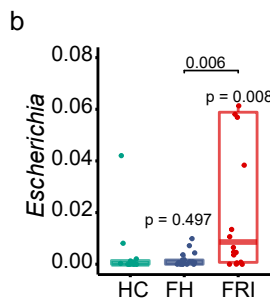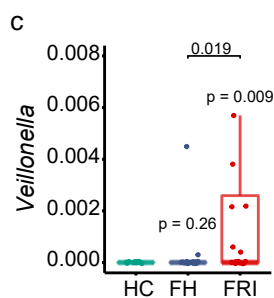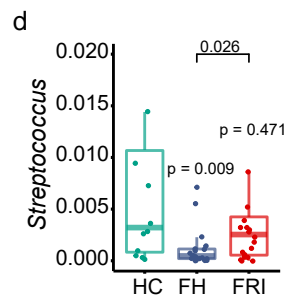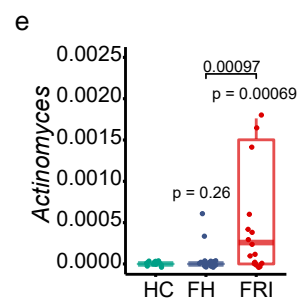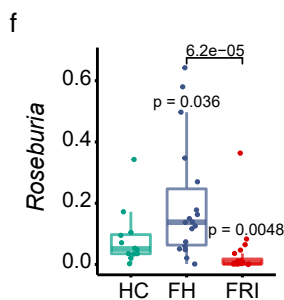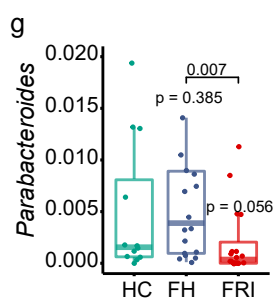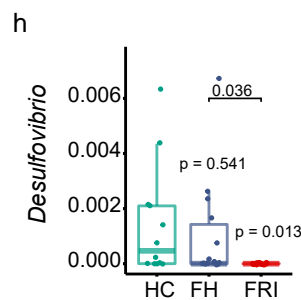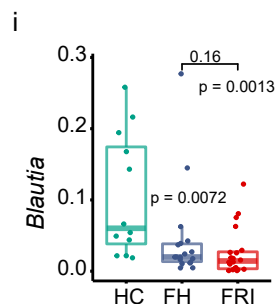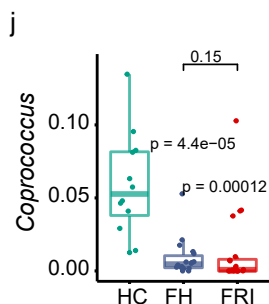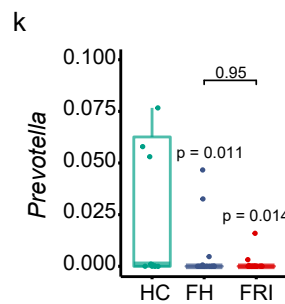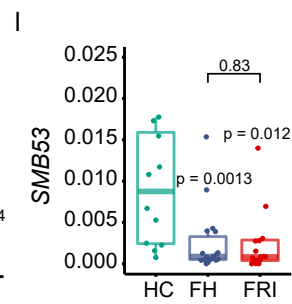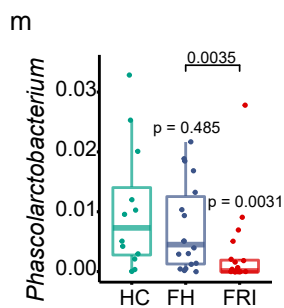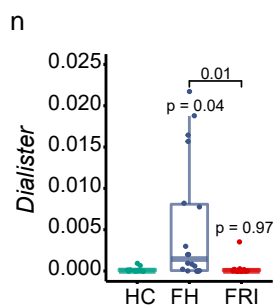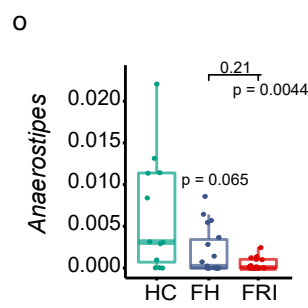

Supplement: Supplementary file 3 [file Image_3.PDF]

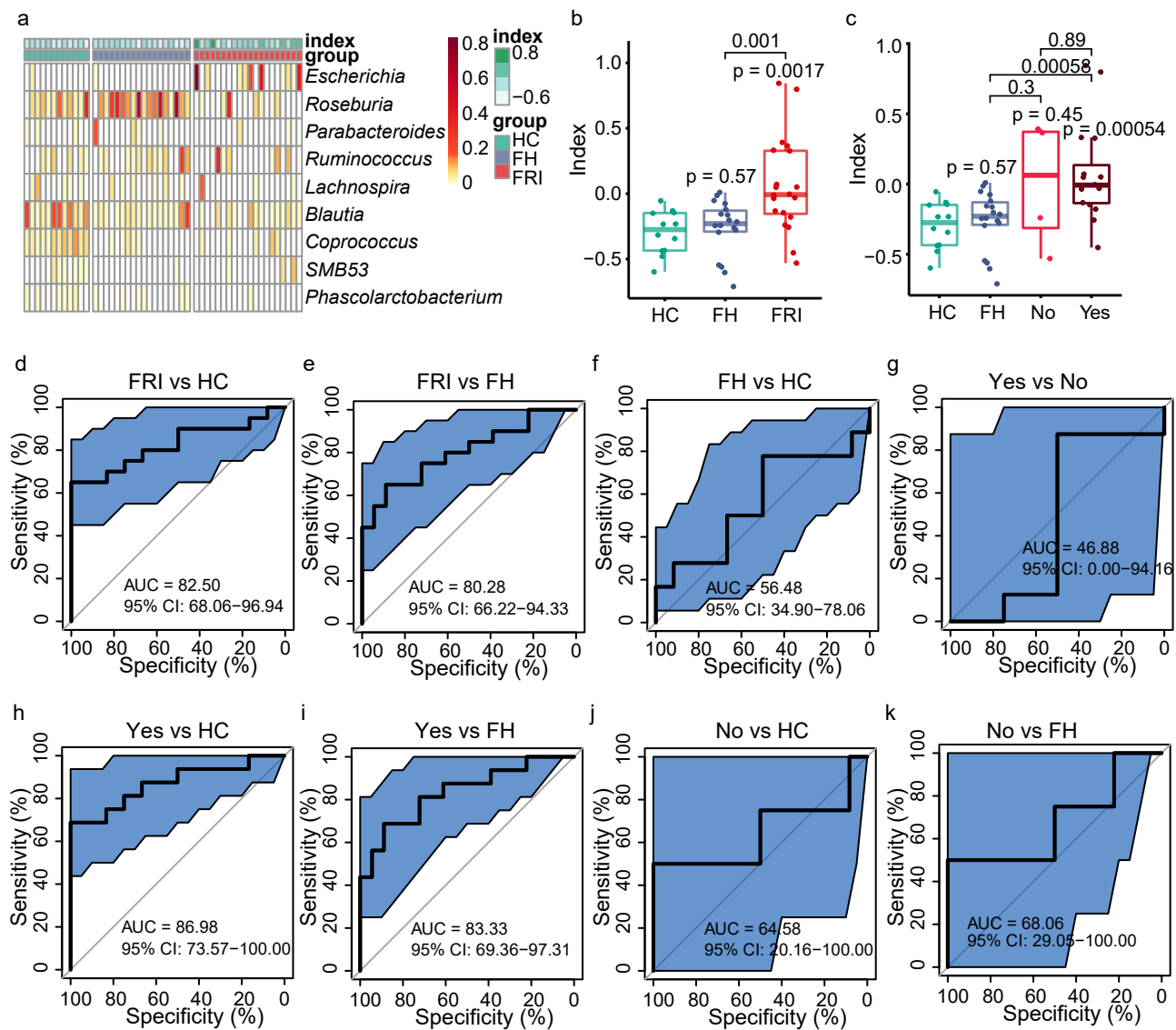

Supplement: Supplementary file 4 [file Image_4.PDF]

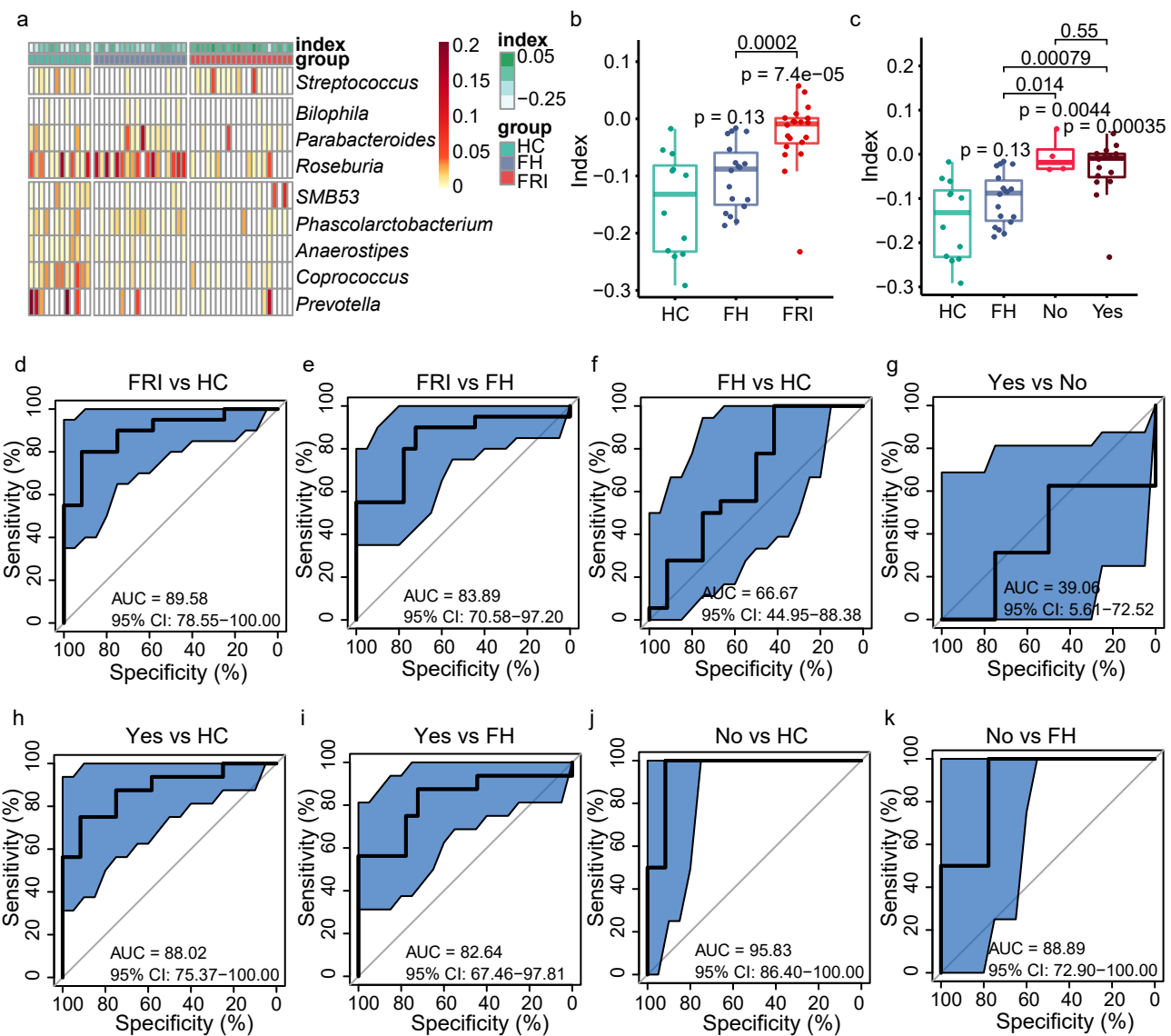

Supplement: Supplementary file 5 [file Image_5.PDF]

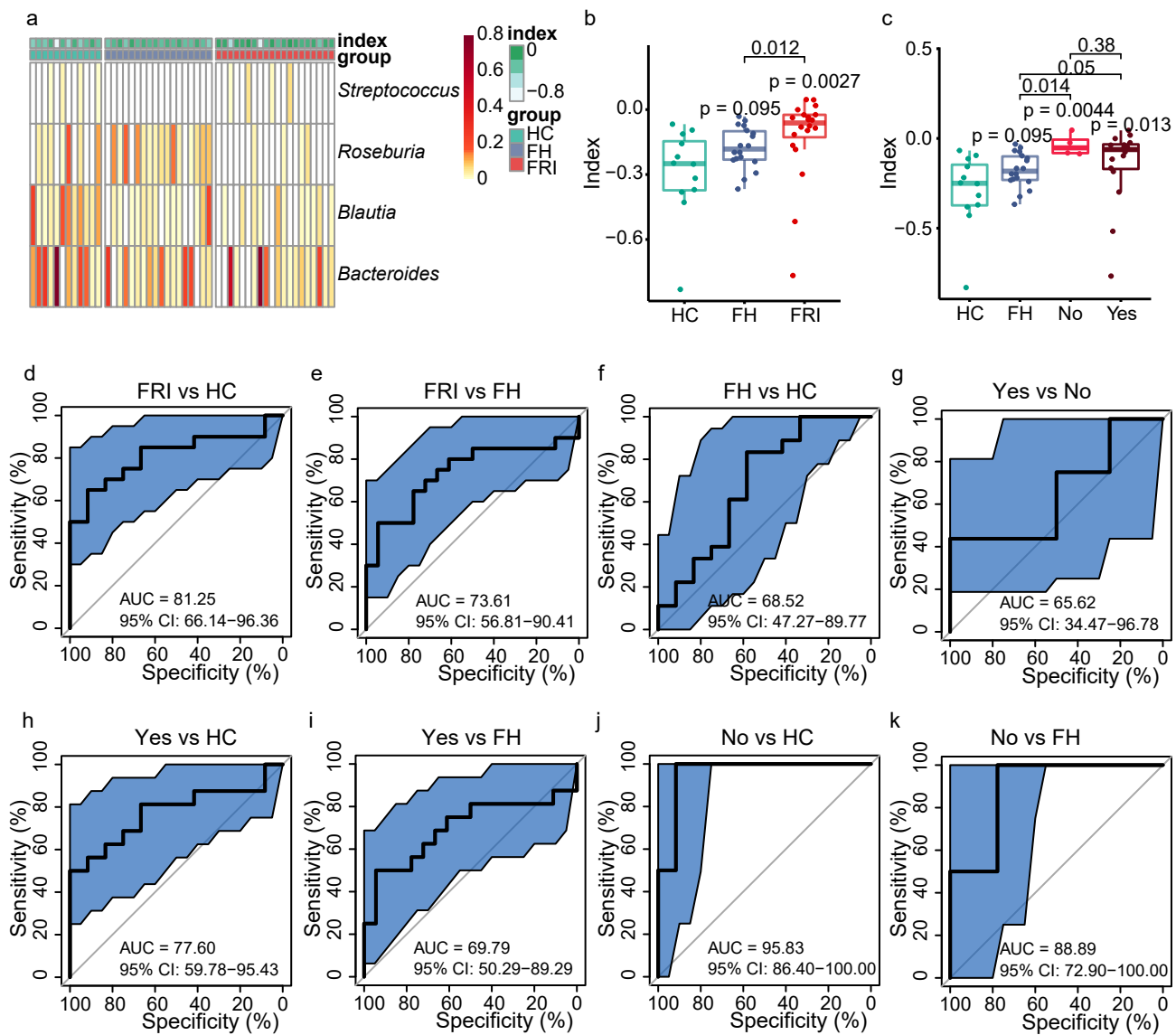

Supplement: Supplementary file 6 [file Image_6.PDF]

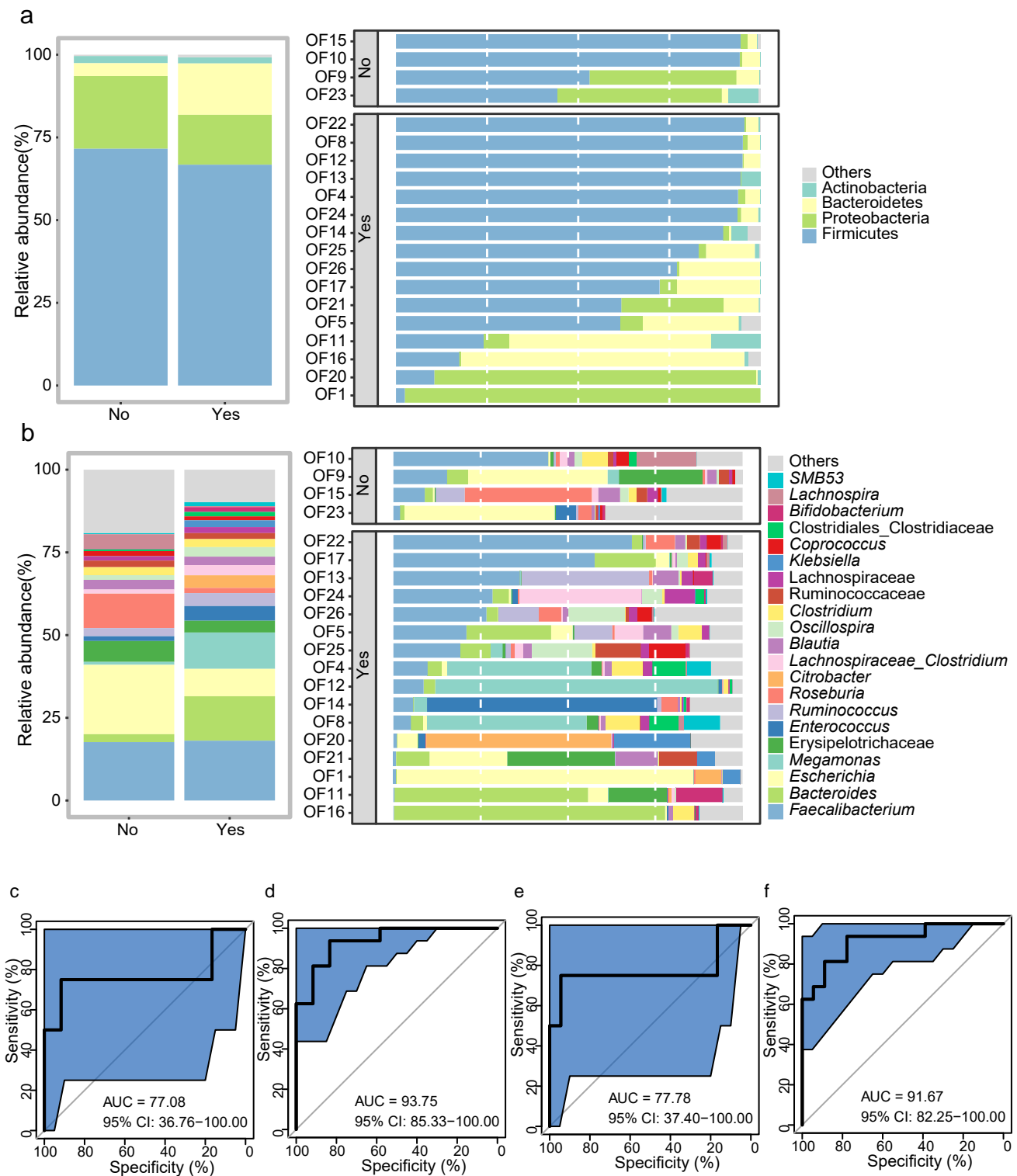

Supplement: Supplementary file 7 [file Image_7.PDF]

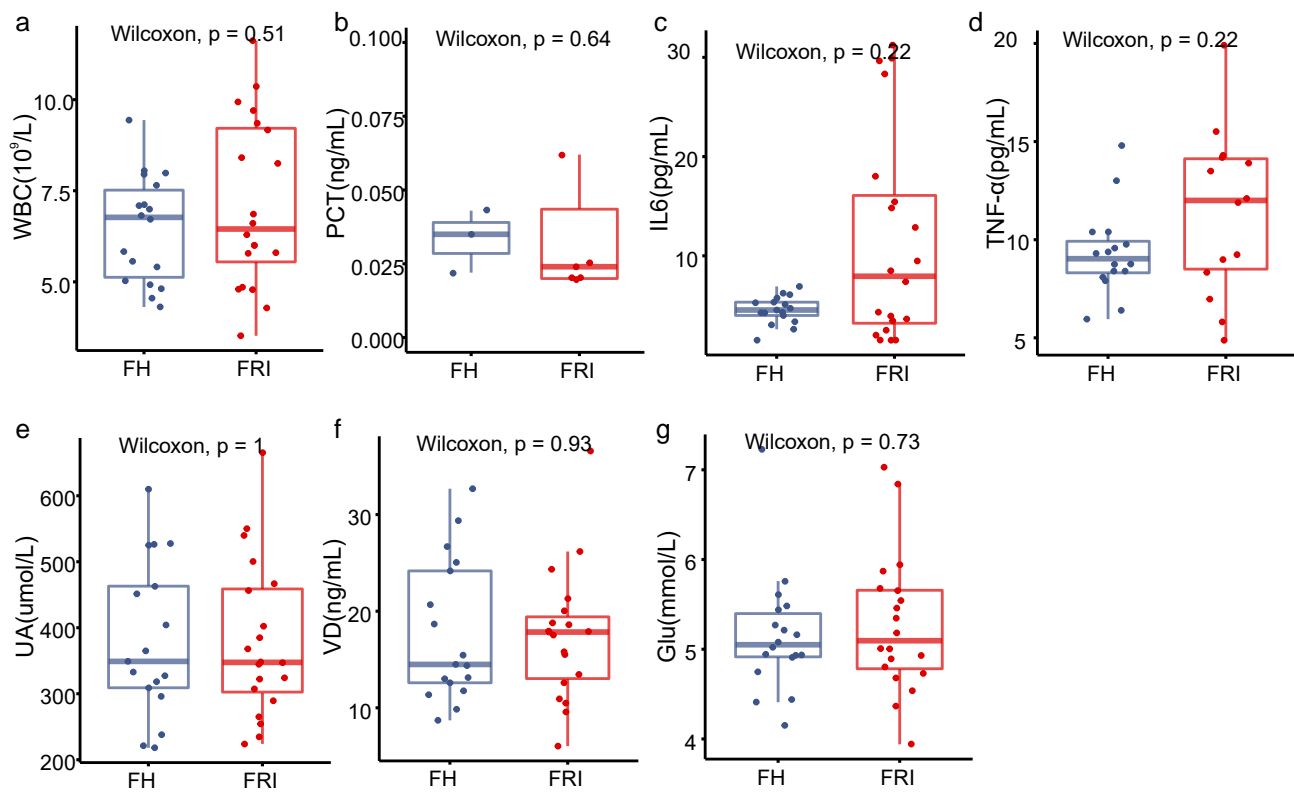

Supplement: Supplementary file 8 [file Image_8.PDF]

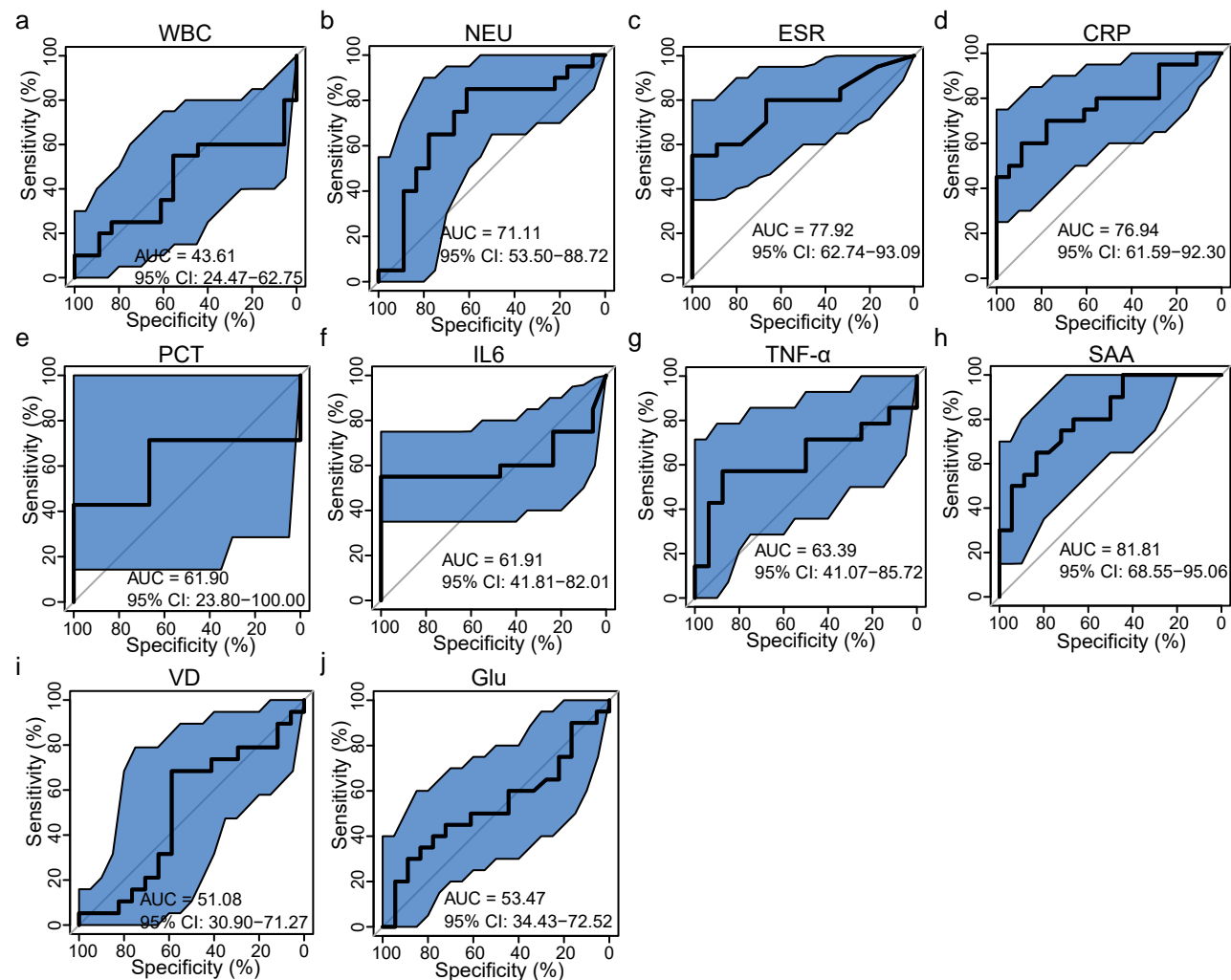

Supplement: Supplementary file 9 [file Image_9.PDF]
