# Supplementary material for: Altered Gut Microbiota as an Auxiliary Diagnostic Indicator for Patients With Fracture-Related Infection
Source: Front Microbiol. 2022 Apr 14;13:723791. doi: 10.3389/fmicb.2022.723791 (PMC9048737; doi:10.3389/fmicb.2022.723791)
Supplement: Supplementary file 11 [file Data_Sheet_1.DOCX]

**Altered gut microbiota as an auxiliary diagnostic indicator for patients with fracture-related infection**

Xingqi Zhao^1, #^, Wenli Tang^2, #^, Haoyang Wan^1, #^, Zixin Lan^3^, Hanjun Qin^1^, Qingrong Lin^1^, Yanjun Hu^1^, Guangchuang Yu^2, *^, Nan Jiang^1, *^, Bin Yu^1, *^

^1^ Division of Orthopaedics and Traumatology, Department of Orthopaedics, Nanfang Hospital, Southern Medical University, Guangzhou, China & Guangdong Provincial Key Laboratory of Bone and Cartilage Regenerative Medicine, Nanfang Hospital, Southern Medical University, Guangzhou, China.

^2^ Department of Bioinformatics, School of Basic Medical Sciences, Southern Medical University, Guangzhou, China

^3^ The Second Clinical Medical College, Southern Medical University, Guangzhou, China.

^#^ These authors contributed equally to this work.

^*^ Co-corresponding author: Address correspondence to Guangchuang Yu, Nan Jiang and Bin Yu, Ph.D., Southern Medical University, No. 1838 North of Guangzhou Ave., Baiyun District, China. E-mail: Guangchuang Yu (gcyu1@smu.edu.cn), Nan Jiang (hnxyjn@smu.edu.cn) and Bin Yu (yubin@smu.edu.cn).

**This file includes:**

**Supplementary Figures S1-9**

**Figure S1.** Gut microbiota composition in FRI, FH, and HC groups. (a) Taxonomy composition at the phylum level. (b) Taxonomy composition at the genus level. (c) Firmicutes to Bacteroidetes ratio.

**Figure S2.** Comparison of bacterial differential abundance between FRI and FH groups. (a) α diversity indicated by Shannon’s diversity index. (b) α diversity indicated by PD whole tree index. (c) PCoA based on Unweighted UniFrac distance. (d) PCoA based on weighted UniFrac distance. (e) LDA score of LEfSe (FRI vs. FH).

**Figure S3.** Abundance of bacteria for Dysbiosis Index building. (a) Mean bacterial genera abundance for Dysbiosis Index construction in FRI, FH, and HC groups. (b-o) Bacterial differential genera abundance in FRI, FH, and HC groups.

**Figure S4.** Dysbiosis Index construction based on generalized linear model (GLM). (a) Bacteria genera for Dysbiosis Index building. (b) Dysbiosis Index (FRI vs. FH vs. HC). (c) The Dysbiosis Index (HC vs. FH vs. Yes vs. No). (d)-(k) AUC showing diagnostic accuracy of gut microbiota composition for FRI vs. HC, FRI vs. FH, FH vs. HC, Yes vs. No, Yes vs. HC, Yes vs. FH, No vs. HC, and No vs. FH. “Yes” for FRI subgroup with sinus tract or pus before operation and “No” for FRI subgroup without sinus tract or pus before operation.

**Figure S5.** Dysbiosis Index construction based on differential genus identified by Linear discriminant analysis effect size (LEfSe) with filtered genus constructed by closed-reference OTU picking method and its ability of discriminate FRI from controls. (a) Bacteria genera for Dysbiosis Index building. (b) Dysbiosis Index (FRI vs. FH vs. HC). (c) The Dysbiosis Index (HC vs. FH vs. Yes vs. No). (d)-(k) AUC showing diagnostic accuracy of gut microbiota composition for FRI vs. HC, FRI vs. FH, FH vs. HC, Yes vs. No, Yes vs. HC, Yes vs. FH, No vs. HC, and No vs. FH. “Yes” for FRI subgroup with sinus tract or pus before operation and “No” for FRI subgroup without sinus tract or pus before operation.

**Figure S6.** Dysbiosis Index construction based on differential genus identified by generalized linear model (GLM) with filtered genus constructed by closed-reference OTU picking method and its ability of discriminate FRI from controls. (a) Bacteria genera for Dysbiosis Index building. (b) Dysbiosis Index (FRI vs. FH vs. HC). (c) The Dysbiosis Index (HC vs. FH vs. Yes vs. No). (d)-(k) AUC showing diagnostic accuracy of gut microbiota composition for FRI vs. HC, FRI vs. FH, FH vs. HC, Yes vs. No, Yes vs. HC, Yes vs. FH, No vs. HC, and No vs. FH. “Yes” for FRI subgroup with sinus tract or pus before operation and “No” for FRI subgroup without sinus tract or pus before operation.

**Figure S7.** Faecal microbiota composition in relation to visible pus or sinus tract in FRI group. (a) Taxonomy composition at the phylum level. (b) Taxonomy composition at the genus level. (c-f) AUC showing diagnostic accuracy of gut microbiota composition in judgement of sinus tract or pus before operation in FRI group (c: HC vs. No; d: HC vs. Yes; e: FH vs. No; f: FH vs. Yes). “Yes” for FRI subgroup with sinus tract or pus before operation and “No” for FRI subgroup without sinus tract or pus before operation.

**Figure S8.** Levels of serum inflammatory markers in FRI and FH groups. WBC: white blood cell count; PCT: procalcitonin; IL-6: interleukin- 6; TNF-α: tumor necrosis factor-α; UA: uric acid; VD: vitamin D; Glu: glucose.

**Figure S9.** AUC of serum inflammatory markers as differential diagnosis indicators.

WBC: white blood cell count; NEU: percentage of neutrophils; ESR: erythrocyte sedimentation rate; CRP: C-reactive protein; PCT: procalcitonin; IL-6: interleukin- 6; TNF-α: tumor necrosis factor-α; SAA: serum amyloid A; VD: vitamin D; Glu: glucose.

**Table S1.** Clinical information of included participants.
